# Mutation analysis of genes that control the G1/S cell cycle in melanoma: TP53, CDKN1A, CDKN2A, and CDKN2B

**DOI:** 10.1186/1471-2407-5-36

**Published:** 2005-04-08

**Authors:** José Luis Soto, Carmen M Cabrera, Salvio Serrano, Miguel Ángel López-Nevot

**Affiliations:** 1 Servicio de Análisis Clínicos e Inmunología, Hospital Universitario Virgen de las Nieves, Avenida Fuerzas Armadas N°2, 18014 Granada, Spain; 2 Servicio de Dermatología, Hospital Universitario San Cecilio, 18014 Granada, Spain

## Abstract

**Background:**

The role of genes involved in the control of progression from the G1 to the S phase of the cell cycle in melanoma tumors in not fully known. The aim of our study was to analyse mutations in TP53, CDKN1A, CDKN2A, and CDKN2B genes in melanoma tumors and melanoma cell lines

**Methods:**

We analysed 39 primary and metastatic melanomas and 9 melanoma cell lines by single-stranded conformational polymorphism (SSCP).

**Results:**

The single-stranded technique showed heterozygous defects in the TP53 gene in 8 of 39 (20.5%) melanoma tumors: three new single point mutations in intronic sequences (introns 1 and 2) and exon 10, and three new single nucleotide polymorphisms located in introns 1 and 2 (C to T transition at position 11701 in intron 1; C insertion at position 11818 in intron 2; and C insertion at position 11875 in intron 2). One melanoma tumor exhibited two heterozygous alterations in the CDKN2A exon 1 one of which was novel (stop codon, and missense mutation). No defects were found in the remaining genes.

**Conclusion:**

These results suggest that these genes are involved in melanoma tumorigenesis, although they may be not the major targets. Other suppressor genes that may be informative of the mechanism of tumorigenesis in skin melanomas should be studied.

## Background

The transition from phase G1 to S of the cell cycle is controlled by sequential activation of cyclin/Cdk complexes (Cyclin-dependent kinases) [1]. Active cyclin/Cdk complexes phosphorylate and inactivate members of the retinoblastoma protein (Rb) family, which are negative regulators of G1 and S-phase progression, leading to the induction of E2F-regulated gene expression and cell proliferation. Inhibitors of cyclin/Cdk complexes, by binding to these complexes, negatively regulate cell cycle progression [2].

Two families of Cdk-inhibitors (CKI) control the actions mediated by cyclin/Cdk complexes. p21 (also called WAF1, and CDKN1A; MIM# 116899) [3] is the founding member of the Cip/Kip family of CKI, which also includes p27 [4] and p57 [5]. Another class of Cdk inhibitors, the so-called INK4 proteins (named for their ability to inhibit cdk4), specifically target the cyclin D-dependent kinases [6]. To date, four INK4 proteins have been identified: the founding member p16^INK4a ^(CDKN2A; MIM# 600160) [7], and three other closely related genes designated p15^INK4b ^(CDKN2B; MIM# 600431) [8], p18^INK4c ^(MIM# 603369) [9] and p19^INK4d ^(MIM# 600927) [9].

In response to irradiation and chemotherapy, p53 protein (MIM# 191170) is stabilised and mediates apoptosis and cell cycle arrest. Whereas the mechanisms of p53-dependent apoptosis are not well understood, p53-dependent cell cycle arrest is known to be primarily mediated by p21, a potent inhibitor of cyclin-dependent kinases that is transactivated by p53 and p73 [10]. In addition to p21, several other cell cycle regulators are induced by p53, such as GADD45 and members of the 14-3-3 family [11].

The TP53 suppressor gene and Cdk-inhibitors such as CDKN1A, CDKN2A, and CDKN2B are targets of tumoral process in different types of tumors [12,13]. Mutations in the TP53 gene occur frequently in skin tumors as basal cell carcinoma (BCC) and squamous cell carcinoma (SCC) [14]. In human melanoma, TP53 mutations are apparently not commonly detected [15,16], and consist mainly of C to T transitions located on dipyrimidine sites originated by UV radiation [17]. In contrast, CDKN2A is deleted or mutated in human sporadic melanomas and derived cell lines [18], and it appears to be the predisposing mutation in some familial melanoma kindreds [19]. A low incidence of mutations has been described for the CDKN2B gene in sporadic melanoma tumors [20]; however, no structural defects have been detected in the CDKN1A gene in human melanoma.

In order to investigate the role of the genes involved in the control of G1/S phase cell cycle progression in human melanomas, the aim of our study was to determine the presence of mutations in TP53, CDKN1A, CDKN2A and CDKN2B genes in primary and metastatic melanomas and melanoma cell lines.

## Methods

### Tumor samples

Thirty-nine specimens of skin melanoma were obtained from the Department of Surgery at the Hospital Universitario San Cecilio of Granada, Spain (Table 1). Melanoma tumors were dissected from normal tissues in fresh samples under sterile conditions, and tumor tissues were frozen in liquid nitrogen and stored at -80°C until DNA isolation. DNA was obtained from peripheral blood from each patient. The following 9 melanoma cell lines were included in this study: MZ2-MEL, MEL-3.0, MEL-2.2, and Mi-13443 were provided by Dr. T. Boon (Ludwig Institute of Cancer Research, Brussels, Belgium); and M31-L, M42-L, M52-L, M34-L, and M59-L were established in our laboratory as described previously [21]. The clinical and pathological characteristics of primary melanoma tumors and derived metastases are described in Table 1. Of the 39 tumors studied, 14 were primary (36%) while the rest were metastatic (18 ganlionar metastases and 4 subcutaneous metastases).

### DNA isolation

DNA was isolated from tumor samples and peripheral blood lymphocytes with the MicroTurboGen Genomic DNA Isolation Kit (Invitrogen, San Diego, California) and the Quiagen kit (Wetsburg, Leusden, The Netherlands) respectively.

### Mutation analysis of TP53, CDKN1A, CDKN2A, and CDKN2B genes

Point mutations were detected by changes in single-stranded conformational polymorphism (SSCP) of DNA amplified by polymerase chain reaction (PCR), as described by Orita et al [22] with slight modifications [23]. TP53 exons 2–11, CDKN1A exon 2, CDKN2B exon 2, and CDKN2A exons 1–2 were amplified. The sequences of the primers used and fragments (bp) amplified are described in Table 2. All TP53 primers used were provided by Clontech (Human p53 Amplier Panels, Palo Alto, CA). A portion of TP53 intron 1, exon 2, intron 2 and exon 3 was amplified using the primers PU2 (sense) and PD3 (antisense). CDKN1A exon 2 was amplified in two overlapping fragments with the following primer pairs: p21-L1/p21-R1 and p21-L2/p21-R2 (Table 2).

Amplifications were performed using 100 ng genomic DNA and α^32^P-dCTP (300 Ci/mmol) in a final volume of 25 μl. The PCR conditions for TP53 exons 2–11 were as follows: 35 cycles at 95°C/30 s, 66°C/45 s and 72°C/1.5 min, with a 10 min extension after the last cycle. CDKN1A exon 2, CDKN2B exon 2, and CDKN2A exons 1–2 were amplified under the same PCR conditions: in a touchdown PCR procedure the temperature of the reaction was lowered by 1°C every second cycle from 68°C to 60°C, at which temperature 30 cycles were carried out.

Amplified samples (2.5 μl) were mixed with 9 μl of sequencing stop solution (USB, Cleveland, OH, USA), 1.5 μl of 0.08 N NaOH, and 15 μl of 0.1% SDS, denatured for 10 min at 95°C, and the samples were quickly cooled in dry ice. Samples of 3 μl were loaded onto a 6% non-denaturing acrylamide gel containing 10% glycerol, and run at room temperature for 4 h at 22 W. Gels were dried at 80°C under vacuum and exposed to x-ray films for 4–16 h.

### DNA sequencing

Asymmetric PCR reactions were purified from agarose gels and reamplified. PCR products were cloned in the PCR 4-TOPO vector using the TOPO TA Cloning Kit for sequencing (Invitrogen, Groningen, The Netherlands). After transformation several clones were picked and sequenced. Sequence analysis was carried out with the Sequenase DNA Sequencing kit (USB), using α^35^S-dATP (DuPont-NEN, Boston, MA) incorporation. Aliquots of the reaction mixture were run on a 6% denaturing acrylamide gel.

## Results

### Intronic single nucleotide polymorphisms and heterozygous point mutations in the TP53 gene

Of a total of 39 melanoma tumors and 9 melanoma cell lines studied by PCR-SSCP, we detected defects in the TP53 gene in 8 of 39 (20.5%) melanoma tumors, and did not find any alteration in melanoma cell lines (Table 3). Mutation analysis showed three novel heterozygous single point mutations in the TP53 sequence and four different single nucleotide polymorphisms, three of which have not been described to date. All novel single point mutations and polymorphisms were compared with the IARC (International Agency for Research on Cancer) TP53 Mutation Database .

**Figure 1:**
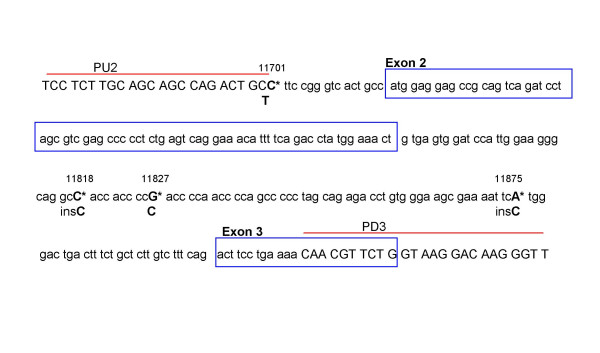
Single nucleotide polymorphisms detected in intron 1 and intron 2 sequences of the TP53 gene in melanoma tumors. Each polymorphism and its positions are indicated by an asterisk (*), to determinate the presence of the polymorphisms the DNA sequence from melanoma tumors and autologous PBLs were compared with control PBLs.

**Figure 2:**
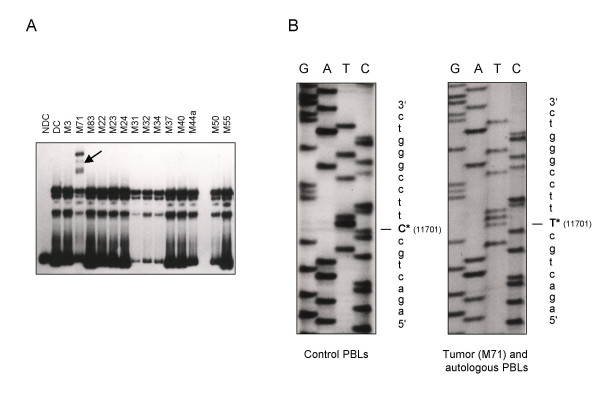
New single nucleotide polymorphism found in intron 1 of the TP53 gene (C>T transition) at position 11701. **(A) **PCR-SSCP analysis of melanoma tumors. The arrow indicates the shifted band in melanoma tumor M71. DC, denaturing control; NDC, non-denaturing control. **(B) **DNA sequence of melanoma tumor M71 and autologous PBLs showing the C to T base change (shown by an asterisk *) at position 11701 compared with control PBLs.

The G to C transversion found at position 11827 in TP53 intron 2 in M4, M7, M38 and M53 melanoma tumors was previously described by Oliva et al [24] (Figure 1). In this study, we found 3 new single nucleotide polymorphisms located at intron 1 and 2 of the TP53 gene when we compared genomic DNA from melanoma tumors and DNA from autologous PBLs with control PBLs (Figure 1). The C to T transition was found at position 11701 of TP53 intron 1 in melanoma tumors M53 and M71 (Figure 2); a C insertion was found at position 11818 of TP53 intron 2 in melanoma tumors M7, M42, M53 and M71; and a C insertion was found at position 11875 of TP53 intron 2 in melanoma tumors M7, M38, M42, M53, and M71 (Table 3).

A heterozygous C deletion in TP53 exon 10 at position 172628 produced a stop codon and truncated the p53 protein in melanoma tumor M34. Melanoma tumors M42 and M43 showed heterozygous single point mutations at TP53 introns 1 and 2. The C to T transition at position 11701 in intron 1, observed in melanoma tumor M42, contrasted with the absence of this transition in autologous PBLs. In contrast, a C insertion was found at position 11818 in intron 2 of TP53 in autologous PBLs, but this polymorphism was not seen in melanoma tumor M43 (Tale 3).

### Mutation analysis of CDKN1A, CDKN2A, and CDKN2B Cdk (Cyclin-dependent kinases) inhibitors genes

Two heterozygous alterations in CDKN2A exon 1 were observed in melanoma tumor M13 one of which novel, whereas no defects were seen in the CDKN1A and CDKN2B genes. The G to A transition produced a stop codon at position 149 [25]; and the novel T to C transition at position 298 resulted in substitution of proline for leucine (Table 3).

## Discussion

### Polymorphisms versus mutations in the TP53 gene in human melanoma

The major carcinogenic agent in most skin cancers is well established as solar ultraviolet light [26]. This is absorbed in DNA, with the formation of UV-specific dipyrimidine photoproducts. About 50% of all skin cancers exhibit TP53 mutations [17], and these mutations are characterised by a specific signature attributed to the UVB part of the solar spectrum. The impact of UVB radiation can be clearly inferred from the characteristic point mutations in TP53 found in human SCC and BCC, consisting of C to T or CC to TT transitions at dipirymidine sites [27]. These findings contrast with the situation in human melanomas, in which TP53 mutations are not commonly detected. The results of the present study support earlier findings that such mutations are indeed infrequent in this type of tumour. The influence of UVB radiation in these mutations is not clear. TP53 mutations in primary melanoma tumors induced by UVB radiation have been described previously by Zerp et al [16]. However, in the tumors examined in our study we did not find TP53 alterations originated by UVB radiation (Table 3). In contrast, we detected two new mutations located in intronic sequences (C deletion at position 11818 in intron 2, and C to T transition at position 11701 in intron 1) (Table 3) and the novel C deletion at codon 350 of TP53 exon 10 which produced a stop codon and truncated protein. More than 90% of the mutations reported in non-melanoma skin cancers and different types of tumors are clustered between exons 4 and 8 [28]. This region is highly conserved throughout evolution and contains the DNA-binding domain of p53, which is essential for its activity [29]. This contrasts with the trans-activation domain (encodes by exons 2 and 3) and the regulatory region (encodes by exons 9 to 11), where few mutations have been described. Therefore, the TP53 single nucleotide polymorphisms detected in these melanoma tumors appear to be their most frequent characteristic.

At least twelve intronic polymorphisms have been described in the human TP53 gene. These include between others a VNTR (variable number tandem repeat) region [30] and *Hae*III restriction fragment length polymorphism (RFLP) [31] in intron 1, a G to C transversion in intron 2 [24], a 16 bp duplication in intron 3 (5'-gacctggagggctggg-3') [32], a *Msp*I RFLP in intron 6 (G to A transition at 61 bp downstream of exon 6) [33,34], a G to C transversion at 37 bp upstream to exon 7 [35], an *Apa*I RFLP in intron 7 [36], and A to T transversion in intron 10 [37]. The melanoma tumors and melanoma cell lines studied here showed the G to C transversion at position 11827 of TP53 intron 2, previously described by Oliva et al [24] in four melanoma tumors (M4, M7, M38, and M53), and three new single nucleotide polymorphisms: C to T transition at position 11701 of TP53 intron 1; C insertion at position 11818 of TP53 intron 2; and C insertion at position 11875 of TP53 intron 2 (Figure 1). Moreover, we found three new heterozygous single point mutations in the TP53 gene (Table 3), the incidence of mutations detected in the TP53 gene accounted for only 7.7% (3 of 39 melanoma tumors) in contrast to 18% (7 of 39 melanoma tumors) of single nucleotide polymorphisms found in these tumors.

Associations between cancer phenotypes and inherited TP53 intronic polymorphisms have been observed in studies of epithelial cancers including ovarian, breast, colon, thyroid, nasopharyngeal, lung cancer, and thyroid [34,35,38-40]. The frequency of G to C transition at position 11827 in intron 2 of TP53 gene (3 of 39 melanoma tumors, 7.7%) is low compared to the frequency of the A1 allele (G to C transition at position 11827) described previously in Caucasian individuals [41]. The polymorphisms that we detected in these melanoma tumors may play a role in the risk of developing skin melanoma in these patients.

### Alterations in cyclin-Cdk inhibitors: CDKN1A, CDKN2A, and CDKN2B

Our results revealed the low incidence of mutations in cyclin-Cdk inhibitors in both melanoma tumors and melanoma cell lines. We detected only two mutations in exon 1 of the CDKN2A gene, both in melanoma tumor M13: G to A transition at position 149 producing a stop codon [25], and the novel missense mutation Leu^298 ^(leucine → proline). The low incidence of CDKN2A mutations found in primary melanomas is concordant with previous reports [42,43]. In contrast, melanomas cell lines show a high incidence of mutations in CDKN2A, with homozygous deletion being the main mechanism of alteration [42]. These results suggest that in sporadic melanoma tumors, CDKN2A might not be a target of mutation, whereas in familial melanoma this mutation accounts for approximately 10% of all cases of tumors.

## Conclusion

We conclude that although none of the cell cycle regulators analysed here can be singled out as a main mutation target for melanoma tumorigenesis, G1/S checkpoint defects are one of the significant factors in the development of melanoma tumors. However, this influence appear to be low in tumors, unlike the situation in melanoma cell lines. Other suppressor genes will be investigated to identify the main targets in the pathogenesis of melanoma.

## Competing interests

The author(s) declare that they have no competing interests.

## Authors' contributions

JLS carried out the molecular genetic studies of SSCP and DNA sequencing. CMC participated in the manuscript drafted. SS participated in the design of the study. MALN participated in the design, coordination and helped to draft the manuscript. All authors read and approved the final manuscript.

## Pre-publication history

The pre-publication history for this paper can be accessed here:



## Figures and Tables

**Table 1:** Melanoma tumor samples

**Tumor**	**^a^Histopathology**	**^b^Breslow (mm)**	**Clark**	**^c^Tumor sample**
M3	SSM	5.5	IV	P
M4a	NM	-	-	Sm
M4b	NM	-	-	Sm
M5	-	-	-	P
M6	-	-	-	Nm
M7a	SSM	4	IV	Nm
M7b	SSM	4	IV	Sm
M8	SSM	0.5	II	P
M13	SSM	3.9	III	P
M18	NM	5	V	Nm
M19	-	-	-	Nm
M21	SSM	3.5	III	P
M22	-	-	-	Nm
M23	SSM	9	IV	Nm
M24	ALM	-	-	Nm
M31	SSM	3	IV	Nm
M32	-	-	-	Nm
M34	SSM	16	V	P
M37	SSM	1.8	III	Nm
M38	NM	9	V	P
M40	NM	3.4	IV	Nm
M42	NM	1.5	IV	Nm
M43	SSM	2.5	IV	P
M44a	NM	10	IV	P
M44b	NM	10	IV	Sm
M45	-	-	-	Nm
M46	-	-	-	P
M49	-	-	-	P
M50	-	-	-	Nm
M52	LMM	-	-	Nm
M53	-	-	-	Nm
M55	ALM	-	V	Nm
M56	LMM	1	III	P
M59	NM	10.1	III	P
M60	SSM	3	III	P
M71	NM	-	-	P
M72	SSM	0.6	III	P
M73	SSM	2.5	III	P
M74	-	-	-	Nm

^a^SSM (superficial spreading melanoma), NM (nodular melanoma), LMM (lentigo maligna melanoma), ALM (acral lentigo melanoma). ^b^Breslow vertical tumor thickness. ^c^P, primary melanoma; Nm, lymph node metastases; Sm, subcutaneous metastases.

**Table 2:** Oligonucleotide primer sequences

**TP53, exons 2 to 11 (GeneBank Accession: X54156)**			
**Exon**	**Primer**	**Sequence 5'→3'**	**Fragment (bp)**
			
	PU2 (sense)	TCCTCTTGCAGCAGCCAGACTGC	
	PD3 (antisense)	AACCCTTGTCCTTACCAGAACGTTG	
			
4	PU4 (sense)	CACCCATCTACAGTCCCCCTTGC	307
	PD4 (antisense)	CTCAGGGCAACTGACCGTGCAAG	
			
5	PU5 (sense)	CTCTTCCTACAGTACTCCCCTGC	211
	PD5 (antisense)	GCCCCAGCTGCTCACCATCGCTA	
			
6	PU6 (sense)	GATTGCTCTTAGGTCTGGCCCCTC	182
	PD6 (antisense)	GGCCACTGACAACCACCCTTAACC	
			
7	PU7 (sense)	GTGTTATCTCCTAGGTTGGCTCTG	139
	PD7(antisense)	CAAGTGGCTCCTGACCTGGAGTC	
			
8	PU8 (sense)	ACCTGATTTCCTTACTGCCTCTTGC	200
	PD8 (antisense)	GTCCTGCTTGCTTACCTCGCTTAC	
			
9	PU9 (sense)	GCCTCTTTCCTAGCACTGCCCAAC	102
	PD9 (antisense)	CCCAAGACTTAGTACCTGAAGGGTG	
			
10	PU10 (sense)	TGTTGCTGCAGATCCGTGGGCGT	130
	PD10 (antisense)	GAGGTCACTCACCTGGAGTGAGC	
			
11	PU11 (sense)	TGTGATGTCATCTCTCCTCCCTGC	153
	PD11 (antisense)	GGCTGTCAGTGGGGAACAAGAAGT	

			
**CDKN1A, exon 2 (GeneBank Accession: AF497972)**			
			
	p21-L1 (sense)	GATGTCCGTCAGAACCCATG	258
	p21-R1 (antisense)	TGCCTCCTCCCAACTCAT	
			
	p21-L2 (sense)	ATGAGTTGGGAGGAGGCA	181
	p21-R2 (antisense)	ATGCTGGTCTGCCGCCGTT	

			
**CDKN2A, exons 1(GeneBank Accession: U12818) and 2 (GeneBank Accession: U12819)**			
			
1	MTS1-L1 (sense)	GAAGAAAGAGGAGGGGCTG	340
	MTS1R1 (antisense)	GCGCTACCTGATTCCAATTC	
2	p16-L2 (sense)	GTCATGATGATGGGCAGC	307
	p16-R2 (antisense)	CTGAGGGACCTTCCGCG	

			
**CDKN2B, exon 2 (GeneBank Accession: AF513858)**			
			
	MTS2-L2 (sense)	TAAGTTTAACCTGAAGGTGG	500
	MTS2-R (antisense)	GGGTGGGAAATTGGGTAAG	

**Table 3:** Single nucleotide polymorphisms (SNPs) and mutations (M) in TP53, CDKN1A, CDKN2A, and CDKN2B genes

**TP53**	
	
Tumor	
M4	11827G>C (intron 2) (SNP)
	11875insC (intron 2) (SNP)
	
M7	11827G>C (intron 2) (SNP)
	11818insC (intron 2) (SNP)
	11875insC (intron 2) (SNP)
	
M38	11827G>C (intron 2) (SNP)
	11875insC (intron 2) (SNP)
	
M42	11701C>T (intron 1) (M)
	11818insC (intron 2) (SNP)
	11875insC (intron 2) (SNP)
	
M43	11875insC (intron 2) (SNP)
	11818delC (intron 2) (M)
	
M53	11701C>T (intron 1) (SNP)
	11827G>C (intron 2) (SNP)
	11818insC (intron 2) (SNP)
	11875insC (intron 2) (SNP)
	
M71	11701C>T (intron 1) (SNP)
	11818insC (intron 2) (SNP)
	11875insC (intron 2) (SNP)
	
M34	17628delC (codon 350: CTC→CTA, exon 10), stop codon at the beginning of exon 11 (M)
	
**CDKN2A, exon 1**	
M13	g.149G>A, TGG (Trp)→TGA (stop codon) (M)
	g.298T>C, CTC (Leu)→CCC (Pro) (M)

	
**CDKN1A, and CDKN2B **No alterations found	
